# Estrogen-related receptor alpha mitigates radiation-induced bowel injury through gut enrichment of *Bacteroides vulgatus*

**DOI:** 10.1080/19490976.2025.2541020

**Published:** 2025-08-19

**Authors:** Seul Gi Shin, June-Young Lee, Jee-Won Choi, Ji-Ho Yoo, In-Chul Jeong, Do-Yeon Kim, Hyun Sik Kim, Seungwha Paik, Gyu-Yong Song, Kyung-Hee Kim, Jin-Man Kim, Jin-Woo Bae, Eun-Kyeong Jo, Sup Kim

**Affiliations:** a Department of Microbiology, Chungnam National University College of Medicine, Daejeon, South Korea; b Department of Medical Science, Chungnam National University College of Medicine, Daejeon, South Korea; c System Network Inflammation Control Research Center, Chungnam National University College of Medicine, Daejeon, South Korea; d Department of Biology, Kyung Hee University, Seoul, South Korea; e Department of Biomedical and Pharmaceutical Sciences, Kyung Hee University, Seoul, South Korea; f College of Pharmacy, Chungnam National University, Daejeon, South Korea; g Department of Pathology, Chungnam National University School of Medicine, Daejeon, South Korea; h Department of Radiation Oncology, Chungnam National University School of Medicine, Daejeon, South Korea

**Keywords:** ESRRA, radiation-induced gastrointestinal toxicity, *Bacteroides vulgatus*, intestinal homeostasis

## Abstract

Radiation-induced gastrointestinal (GI) toxicity can be a major cause of morbidity in patients undergoing abdominal radiotherapy. There is an unmet need for treatments to ameliorate GI toxicity. Estrogen-related receptor alpha (ESRRA), a protein involved in the regulation of inflammation and autophagy, is widely expressed across human tissues. Our recent findings on ESRRA’s significant contribution to intestinal homeostasis and inflammation control in inflammatory bowel disease inspired us to investigate its potential role in radiation-induced gastrointestinal injury. *esrra*^*-/-*^ mice showed distinct gut microbiota composition and increased susceptibility to abdominal irradiation with significant alteration of microbiota and increased intestinal inflammation. *B. vulgatus* reversed gut pathology in *esrra*^*-/-*^ mice by improving intestinal barrier function, reducing inflammation, and restoring the expression of *Tfeb* and its downstream genes. Additionally, in patients treated with abdominal radiotherapy, decreased ESRRA expression in rectal tissues correlated with increased IL-6 expression and radiation induced diarrhea. Our findings indicate that ESRRA contributes to intestinal homeostasis through gut enrichment of *B. vulgatus*.

## Introduction

The gastrointestinal (GI) system has rapidly proliferating cells, which make it one of the most radiosensitive organs in the body.^1,2^ As a result, cancer patients receiving abdominal radiotherapy and individuals exposed to accidental irradiation experienced gastrointestinal toxicity during and after the event.^1,2^ The most common toxicity symptoms observed in patients were nausea, vomiting, diarrhea, and abdominal pain, all of which significantly reduce quality of life.^2–5^ Moreover, this condition can limit the ability to deliver the tumoricidal dose necessary for sterilizing abdominal and pelvic tumors.^6^ Therefore, Consequently, GI toxicity remains a significant concern after radiation exposure, with growing evidence emphasizing its implications for healthcare systems and patient outcomes.^7,8^

Although the advent in radiation therapy treatment planning and delivery technologies such as intensity-modulated radiotherapy (IMRT) and image-guided radiotherapy (IGRT) has significantly reduced adverse effects, radiation-induced GI toxicity remains substantial clinical challenges.^9^ During radiotherapy, exposure to ionizing radiation can injure adjacent normal intestinal tissues, resulting in generation of reactive oxygen species, production of pro-inflammatory cytokines and activated macrophages, and role of bone marrow-derived progenitor and stem cells.^10^ Better understanding the molecular mechanisms mediating oxidative stress and inflammation may provide novel insight on the pathophysiology of radiation-induced injury of intestinal tissues. This knowledge will facilitate the identification of molecular targets, paving the way for novel therapeutic strategies to improve treatment outcomes.

Estrogen-related receptor alpha (ESRRA), a member of orphan nuclear receptor transcription factors, has been initially discovered due to its structural similarity to estrogen receptors.^11^ Instead of regulating estrogen pathway, ESRRA primary controls energy metabolism through ligand independent transcriptional action driven by coactivator interactions.^11^ ESRRA is widely expressed in various human tissues and associated with metabolic disorders, liver disease, and breast cancer.^11,12^ .^13–15^ Beyond its conventional roles in metabolism, ESRRA plays a negative regulator of TLR-induced inflammatory responses through inducing *Tnfaip3* transcription and controlling the metabolic reprogramming.^16^ Additionally, ESRRA enhanced the transcriptional activation of numerous autophagy-related genes (*Atg5, Becn1, Atg16l1*) at both transcriptional and post-translational levels, which is essential for regulation of excessive inflammation during mycobacterial infection.^17^ Collectively, these findings highlight ESRRA may play a key function in the regulation of immune homeostasis by maintaining a balanced pro-inflammatory and anti-inflammatory response.

Besides its systemic immune modulation property, there is growing interest in ESRRA’s role in regulating organ-specific immune modulation. Because of abundant expression in intestinal tissues, additional study may reveal previously unappreciated mechanisms regulating intestinal function.^18^ According to recent studies, ESRRA contributes to intestinal homeostasis through autophagy activation and gut microbiota control, dysfunctional mitochondria, preservation of epithelial integrity.^18,19^ Building on these findings, the current research investigated the role of ESRRA in radiation-induced GI injury.

The microbiome, which is composed of the collective genomes of trillions of living microorganisms, forms a complex and essential ecosystem within the human body. Recent research has highlighted the dynamic crosstalk between the microbiota and immune system, which is vital for maintaining health and is implicated in various diseases, including cardiovascular disease, cancer, respiratory disease, and IBD.^20^ There is intense interest in understanding the interactions between hosts and microbiota, with novel microbiota-targeted therapies being explored as potential treatments for various conditions, including colitis.^21^ However, the relationships among the gut microbiota, radiotherapy, and inflammation, as well as their impact on intestinal homeostasis, remain largely unexplored.

In this study, we used whole-abdominal irradiation (WAI) and rectal irradiation mouse models to evaluate the effectiveness of ESRRA in reducing radiation-induced GI toxicity. By monitoring survival rates and analyzing mRNA and protein expression, we investigated the impact of ESRRA on inflammation and mucosal integrity postirradiation. To explore the role of the microbiome, we evaluated the protective effect of *Bacteroides vulgatus*, which is depleted in *esrra*^*-/-*^ mice, using a WAI mouse model.^18^ We also examined the expression of ESRRA and IL-6 in the rectal tissues of patients by comparing nonirradiated and irradiated samples through IHC to confirm the clinical relevance of the findings. By integrating animal models and patient sample analyses, we comprehensively assessed the therapeutic potential of ESRRA. Our results could lead to improved treatment strategies for patients suffering from radiation-induced GI complications.

## Results

### ESRRA is crucial for controlling host susceptibility to radiation-induced gastrointestinal toxicity

**Figure 1.**
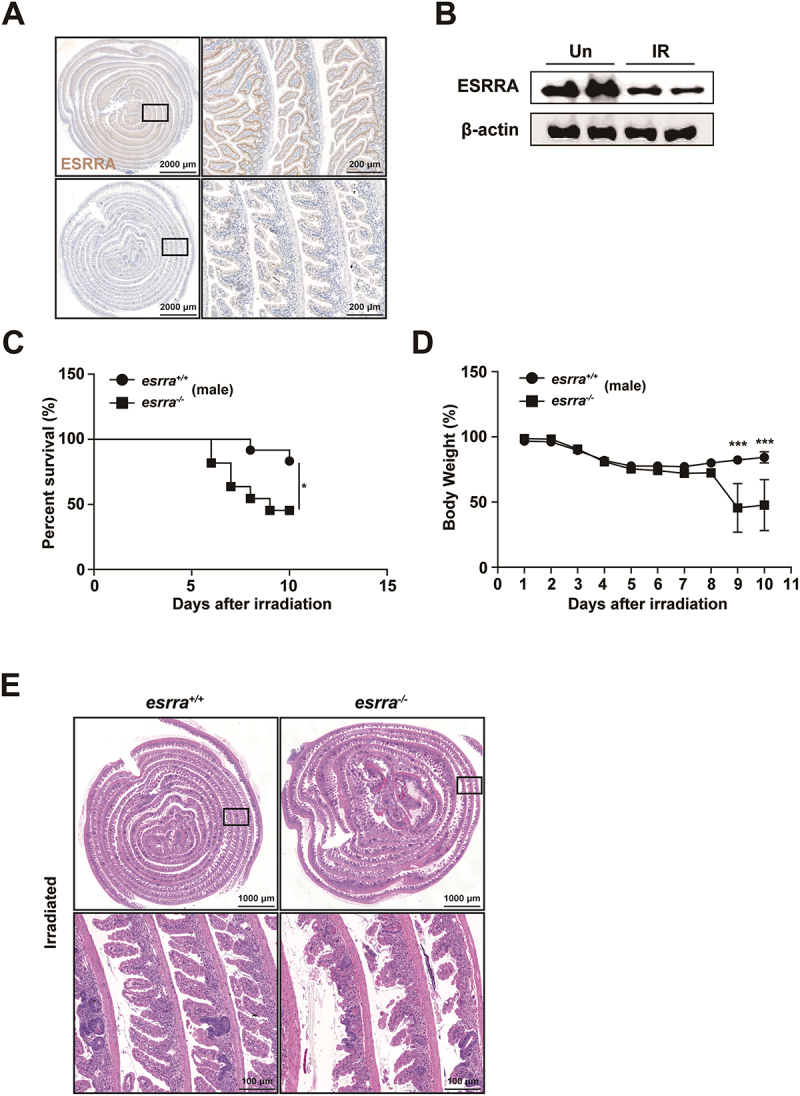
ESRRA expression is critical for protecting against radiation-induced gastrointestinal toxicity. (A) representative immunohistochemistry images showing ESRRA expression in the small intestine of *esrra*^*+/+*^ mice subjected to whole-abdominal irradiation (WAI). Scale bar = 100 µm. (B) ESRRA expression in the small intestine of *esrra*^+/+^ mice by western blotting. (C) survival rates of *esrra*^+/+^ and *esrra*^-/-^ male mice postirradiation with 17 Gy WAI. The data are presented as Kaplan‒Meier survival curves (*n* > 5 per group). (D) body weight changes in *esrra*^+/+^ and *esrra*^-/-^ male mice following 17 Gy WAI (*n* > 5 per group). (E) representative histological images (H&E staining) of small intestine tissues from *esrra*^+/+^ and *esrra*^-/-^ male mice following 17 Gy WAI. Scale bar = 100 µm. *p < 0.05, ***p < 0.001. log-rank (mantel‒Cox) test (C) and two-way ANOVA (D). Tissue samples for immunohistochemistry and H&E staining were prepared using the Swiss roll method. The data are representative of three independent experiments, and the error bars indicate the means ± SEMs. Un, untreated. IR, irradiated.

ESRRA protein expression is significantly downregulated in colon tissues following dextran sodium sulfate (DSS) challenge.^18^ To investigate the role of ESRRA in radiation-induced GI toxicity, we first examined whether its expression pattern changed in a WAI mouse model. Consistent with previous findings, its levels were significantly decreased in irradiated tissues of the small intestine, as shown in Figure 1A,B.

To explore the role of ESRRA in providing protection against irradiation, we exposed *esrra*^*+/+*^ and *esrra*^*-/-*^ mice to 17 Gy of WAI. The survival rate of *esrra*^*-/-*^ mice decreased dramatically after WAI, as shown in Figures 1C and S1A. Age- and sex-matched *esrra*^*+/+*^ mice regained their initial body weight by day 8, whereas *esrra*^*-/-*^ mice did not (Figure 1D and S1B). As depicted in Figure 1D and S1B, the body weight of the *esrra*^*-/-*^ mice was significantly reduced to less than 50% of the initial weight by day 8 post-WAI. Furthermore, the survival rate of *esrra*^*-/-*^ mice dramatically decreased after WAI, as shown in Figure 1C and S1A. Histological evaluation revealed severe intestinal injury in WAI-exposed *esrra*^*-/-*^ mice, characterized by extensive mucosal inflammation and edematous submucosal tissues, compared to the relatively mild damage observed in *esrra*^*+/+*^ mice (Figure 1E). Collectively, these data suggest that the absence of ESRRA increases susceptibility to radiation-induced GI toxicity.

### *Esrra* deletion upregulates inflammatory responses in irradiated tissues of the small intestine

**Figure 2.**
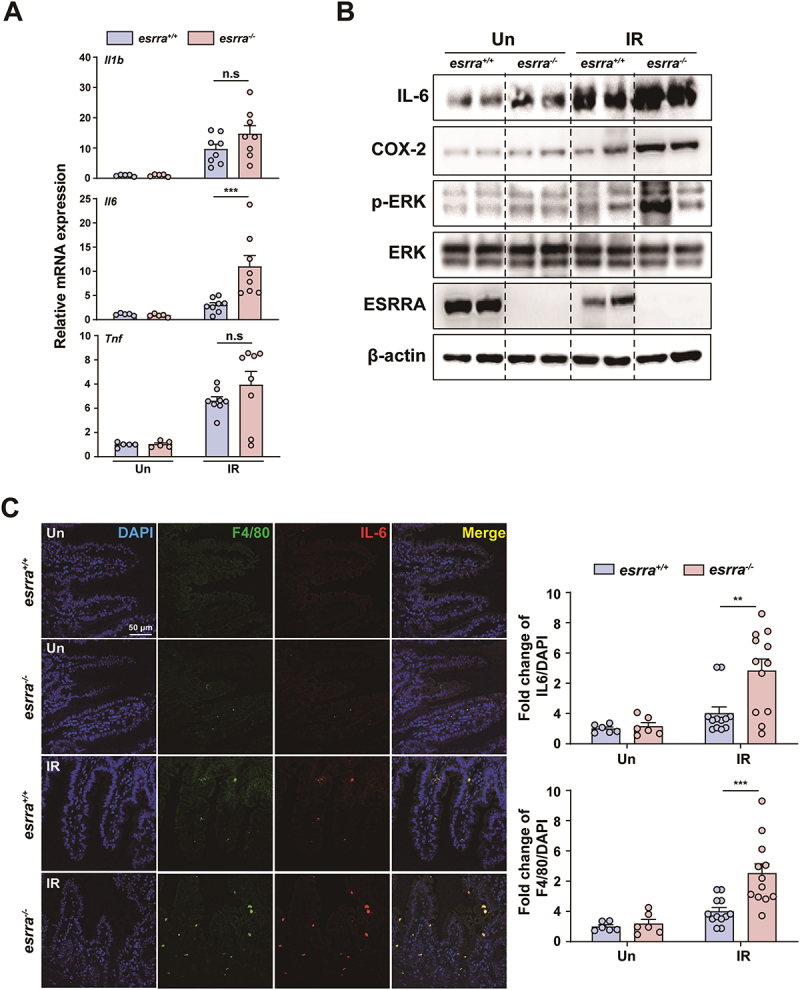
ESRRA deficiency enhances inflammatory responses in irradiated small intestine tissues. (A) quantitative PCR analysis of inflammatory cytokine mRNA levels (*Il1β, Il6, and Tnf*) in mice treated with WAI (0 and 5 days after WAI; *n* > 5 per group). (B and C) Western blot analysis of IL-6, COX-2, phosphorylated ERK, ERK and ESRRA expression in jejunal tissue from unirradiated and irradiated *esrra*^+/+^ and *esrra*^-/-^ mice. β-actin served as a loading control. (D) representative immunofluorescence images and quantitative analysis of the staining showing IL-6 (red) and ADGRE1/F4/80 (green) protein levels in small intestine tissues following 17 Gy WAI. Scale bar = 50 µm. **p < 0.01, ***p < 0.001, n.s.: not significant. Two-way ANOVA (A and C). The experiments were performed at least three times, and the values are presented as the means ± SEMs. Un, untreated. IR, irradiated.

ESRRA plays a critical role in inflammatory responses, the maintenance of mitochondrial function and intestinal permeability.^18^ Therefore, we examined whether *esrra* ablation influences genes involved in the innate immune response, mitochondrial dynamics and mucosal permeability in small intestine tissues. There were no significant differences in the mRNA expression levels of *Il1β, Tnf, or Cxcl2*; however, those of *Il6 and Ccl2* significantly increased in *esrra*^*-/-*^ mice following WAI (Figures 2A and S2A). Among the numerous genes involved in mitochondrial function and the tight junction complex, WAI significantly decreased *Chchd10, Cldn3, Cldn4, and Cldn15* in *esrra*^*-/-*^ mice compared to littermate controls (Figure S2A). Western blotting analysis revealed that the protein levels of COX-2 and phosphorylated ERK (p-ERK) were significantly elevated in irradiated *esrra*^*-/-*^ mice (Figure 2B). Immunofluorescence analyses further demonstrated increased IL-6 protein expression and macrophage infiltration following WAI (Figures 2C and S2B). Specifically, the IL-6 and F4/80 protein expression levels were significantly elevated in the intestines of the *esrra*^*-/-*^ mice (Figure 2C). Consistently, IHC analysis further confirmed increased expression of IL-6 and CD68 in the intestines of the *esrra*^*-/-*^ mice (Figure S2B). These data suggest that *esrra* deficiency leads to increased inflammation in the small intestine, elevated IL-6 expression and enhanced macrophage infiltration.

### ESRRA expression contributes to the radioprotective effects observed in the small intestine of mice

**Figure 3.**
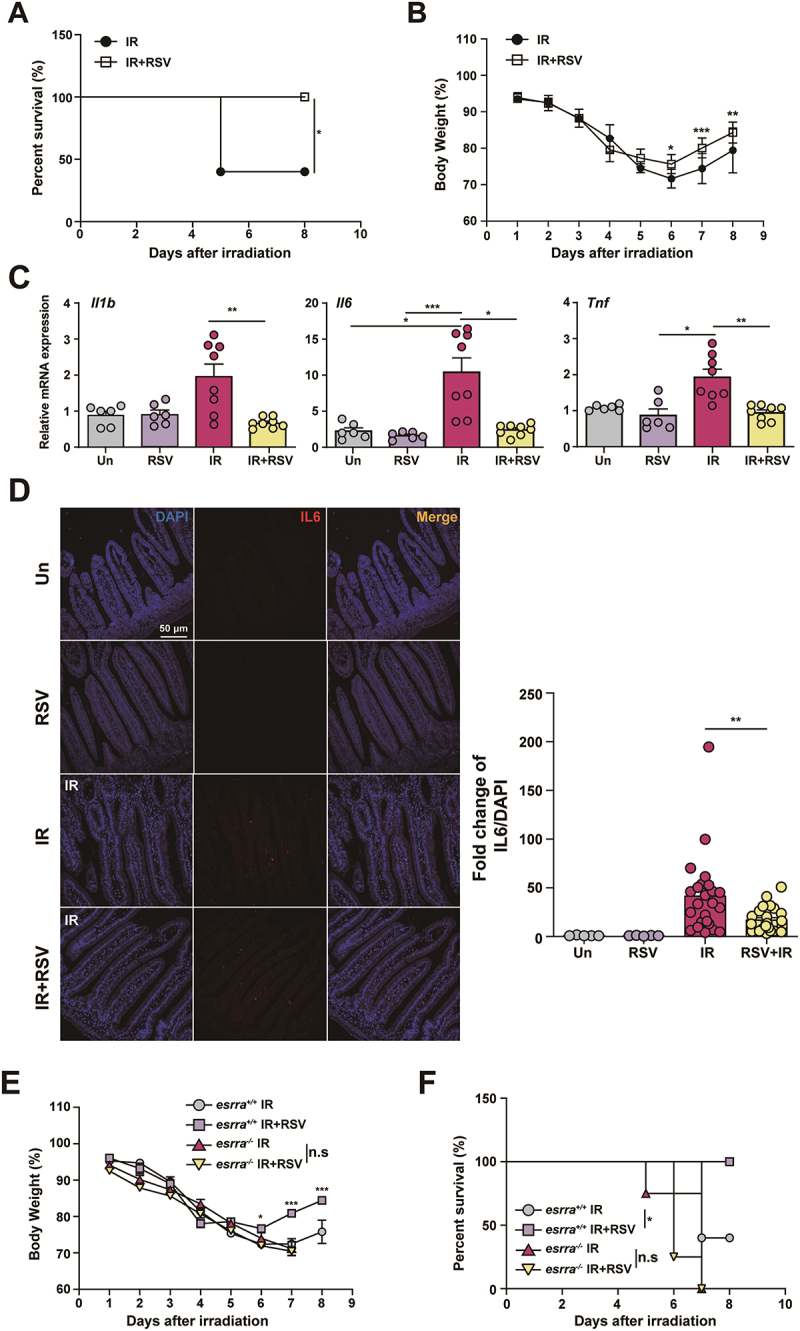
The ESRRA agonist resveratrol mitigated radiation-induced gastrointestinal toxicity. (A) survival rates of *esrra*^+/+^ mice treated with resveratrol (40 mg/kg) or PBS via intraperitoneal (IP) injection on days 3 and 5 following WAI. The data are presented as Kaplan‒Meier survival curves (*n* = 9 per group). (B) body weight changes in *esrra*^+/+^ mice treated with resveratrol (40 mg/kg) or PBS via IP injection on days 3 and 5 following WAI (*n* = 9 per group). (C) quantitative PCR analysis of inflammatory cytokine mRNA levels (*Il1β, Il6, and Tnf*) in mice treated with resveratrol (40 mg/kg) or PBS via IP injection on days 3 and 5 following WAI (*n* > 5 per group). (D) representative immunofluorescence images and quantitative analysis showing IL-6 (red) protein levels in small intestine tissues from resveratrol- or PBS-treated *esrra*^+/+^ mice following 17 Gy WAI. Scale bar = 50 µm. (E) body weight changes in *esrra*^+/+^ and *esrra*^-/-^ mice treated with resveratrol (40 mg/kg) or PBS via IP. injection on days 3 and 5 following WAI. The data are presented as Kaplan‒Meier survival curves (*n* = 5 per group). (F) survival rates of *esrra*^+/+^ and *esrra*^-/-^ mice treated with resveratrol (40 mg/kg) or PBS via intraperitoneal (IP) injection on days 3 and 5 following WAI (*n* = 5 per group). *p < 0.05, **p < 0.01, ***p < 0.001, n.s.: not significant. Log-rank (mantel‒Cox) test (A and F), two-way ANOVA (B and E), and one-way ANOVA (C and D). The data are representative of three independent experiments, and the error bars indicate the means ± SEMs. Un, untreated. IR, irradiated; RSV, resveratrol.

We evaluated the effect of resveratrol, a known ESRRA agonist, on radiation-induced GI toxicity.^22^ Mice received resveratrol (40 mg/kg) via intraperitoneal (IP) injection on days 3 and 5 following WAI. By day 5 postirradiation with 17.5 Gy WAI, survival rates and body weights were notably lower in the WAI-only group than in the resveratrol-treated group (Figures 3A,B). More than half of the mice in the WAI-only group died by day 5 postirradiation, whereas none of the resveratrol-treated mice died (Figure 3A). In addition, quantitative polymerase chain reaction (qPCR) analysis revealed that the mRNA expression levels of *Il1β*, *Il6, and Tnf* were significantly lower in the tissues of resveratrol-treated mice than in those of *esrra*^*+/+*^ mice (Figure 3C). Immunofluorescence analyses revealed decreased IL-6 protein expression and macrophage infiltration in the tissues of resveratrol-treated mice compared to e*srra*^*+/+*^ mice (Figures 3D and S3A). These results indicate that ESRRA agonists may alleviate radiation-induced inflammation and mortality.

To further investigate the influence of resveratrol on *esrra*^*-/-*^ mice and their littermate controls, a subsequent experiment was conducted. Mirroring the previous experiment, compared with those in the other groups, the body weight and survival rates of the resveratrol-treated *essra*^*+/+*^ mice were significantly greater on day 7 post-17 Gy WAI (Figures 3E,F). In contrast, *esrra*^*-/-*^ mice did not exhibit improved survival rates (Figures 3E,F), indicating that ESRRA activation is essential for resveratrol-mediated radioprotection. These findings suggest that ESRRA is both required and sufficient to mediate the intestinal radioprotection elicited by resveratrol treatment.

### *Bacteroides vulgatus* mitigates radiation-induced gut toxicity in *esrra*^-/-^ mice

**Figure 4.**
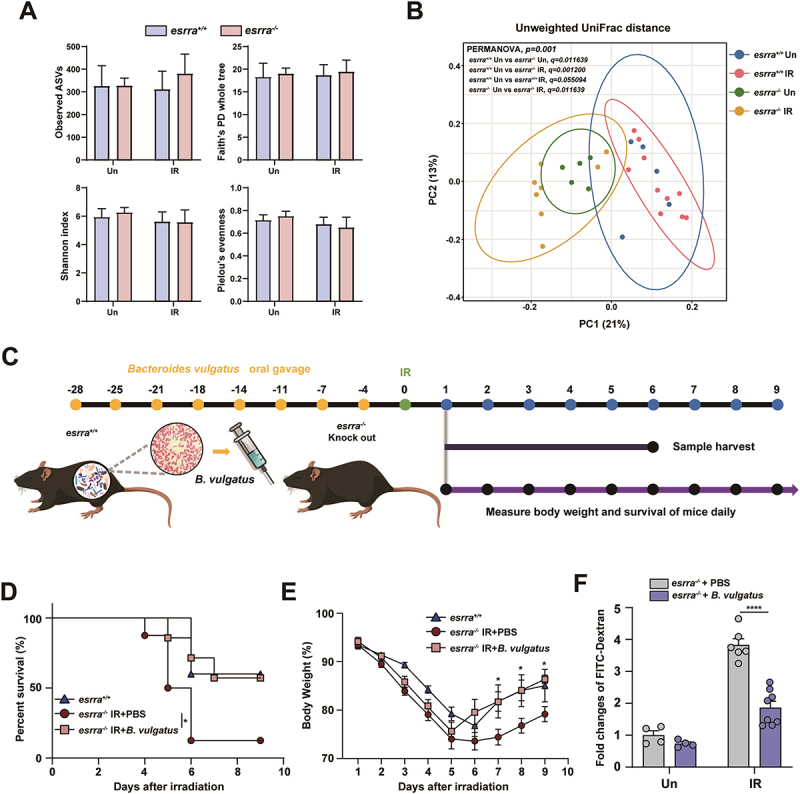
*Bacteroides vulgatus* ameliorates radiation-induced gastrointestinal toxicity in *esrra*^-/-^ mice. (A) alpha diversity results in both *esrra*^+/+^ and *esrra*^-/-^ mice following WAI (0 and 2–3 days after WAI; *n* > 5 per group). (B) the PCoA results based on unweighted UniFrac distances of 16S rRNA amplicon sequencing of the feces from *esrra*^*+/+*^ and *esrra*^*-/-*^ mice before and after WAI, respectively. (C) schematic illustration of the *B. vulgatus* oral gavage experimental timeline. (D) survival rates of *esrra*^-/-^ mice treated with *B. vulgatus* or PBS for 4 weeks before 17 Gy WAI. The data are presented as Kaplan‒Meier survival curves (*n* > 5 per group). (E) body weight changes in *esrra*^-/-^ mice treated with *B. vulgatus* or PBS for 4 weeks before 17 Gy WAI (*n* > 5 per group). (F) serum FITC-dextran levels as a measure of intestinal permeability in *esrra*^-/-^ mice treated with *B. vulgatus* or PBS for 4 weeks before 17 Gy WAI (*n* > 4 per group.) *p < 0.05, ****p < 0.0001. Multiple Mann-Whitney test with Holm-Šídák multiple comparison test (A), PERMANOVA with 1000 permutations with pairwise comparison (B), log-rank (mantel‒Cox) test (D) and two-way ANOVA (E and F). The experiments were performed at least three times, and the values are presented as the means ± SEMs. Un, untreated. IR, irradiated.

Previously, we reported that the gut microbiota was significantly different between *esrra*^*+/+*^ mice and *esrra*^*-/-*^ mice before and after DSS treatment. Notably, *esrra*^*+/+*^ mice have more abundant populations of *Bacteroides* than *esrra*^*-/-*^ mice.^18^ All sequences belonging to the genus *Bacteroides* were annotated using the EZbiocloud database.^23^ Among these, the operational taxonomic units (OTUs) identified as *B*. *vulgatus*, exhibiting over 99% sequence similarity, accounted for the largest proportion of *Bacteroides* species. *B. vulgatus* has been shown to play a protective role against *Escherichia coli*-induced colitis in gnotobiotic *il2* KO mice.^24^ We aimed to confirm whether the gut microbiota differed between *esrra*^*+/+*^ mice and *esrra*^*-/-*^ mice, as observed in prior study, and to examine the abundance of *Bacteroides*. Additionally, we also evaluated the effect of WAI on the gut microbial composition. Alpha diversity metrics such as observed ASVs, Faith’s phylogenetic diversity, Shannon index, and Pielou’s evenness showed no significant differences between *esrra*^*+/+*^ mice and *esrra*^*-/-*^ mice both before and after WAI (Figure 4A). Beta diversity was analyzed using principal coordinate analysis (PCoA) based on both unweighted and weighted UniFrac distance matrices. Significant separation between all experimental groups was observed in PCoA based on unweighted UniFrac distances (PERMANOVA, *p* = 0.0001) (Figure 4B). Pairwise comparison results showed significant differences in gut microbiota were observed between *esrra*^*+/+*^ mice and *esrra*^*-/-*^ mice both before and after WAI (*p* < 0.05). However, while no significant changes in gut microbiota in *esrra*^*+/+*^ mice were identified before and after WAI (*p* > 0.05), significant alterations were detected in *esrra*^*-/-*^ mice post-WAI (*p* < 0.05). Similar results were observed in PCoA based on weighted UniFrac distances, considering bacterial abundance (PERMANOVA, *p* = 0.0001) (Figure S4A). These pairwise comparisons across all pairs further confirmed the significant microbiota changes (*p* < 0.05). These results suggest that WAI induces significant changes in the gut microbiota and the absence of ESRRA alters the gut microbiota, increasing susceptibility to microbial changes induced by WAI.

Consistent with previous findings, *B*. *vulgatus* was detected only in *esrra*^*+/+*^ mice in this data (Figure S4B). To investigate whether *B. vulgatus* can alleviate radiation-induced GI toxicity in mice, we assessed the gut microbiota composition and severity of colitis by monitoring weight, survival, and mRNA expression during the induction period and by measuring intestinal permeability on day 5 post-WAI exposure. Figure 4C shows a schematic of the *B. vulgatus* oral gavage experiment. Briefly, we administered *B. vulgatus* for 4 weeks prior to the WAI. The irradiation experiment was performed 1 week after the administration of *B. vulgatus* or phosphate-buffered saline (PBS). The gut microbial compositions of the experimental groups were visualized using relative abundance bar chart at the genus level for each sample (Figure S5A). Additionally, we quantified the abundance of *B*. *vulgatus* across all experimental groups using real-time qPCR with *B*. *vulgatus* specific primer.^25^ The *esrra*^*+/+*^ mice showed a significantly higher *B*. *vulgatus* abundance compared to *esrra*^*-/-*^ mice. Following *B*. *vulgatus* treatment, e*srra*^*-/-*^ mice exhibited an increasing trend in *B. vulgatus* abundance compared to the PBS-treated group (Figure S5B). Also, the survival rates and weight loss in the *B. vulgatus*-treated group were significantly greater than PBS-treated group (*p* < 0.05; Figure 4D,E).

**Figure 5.**
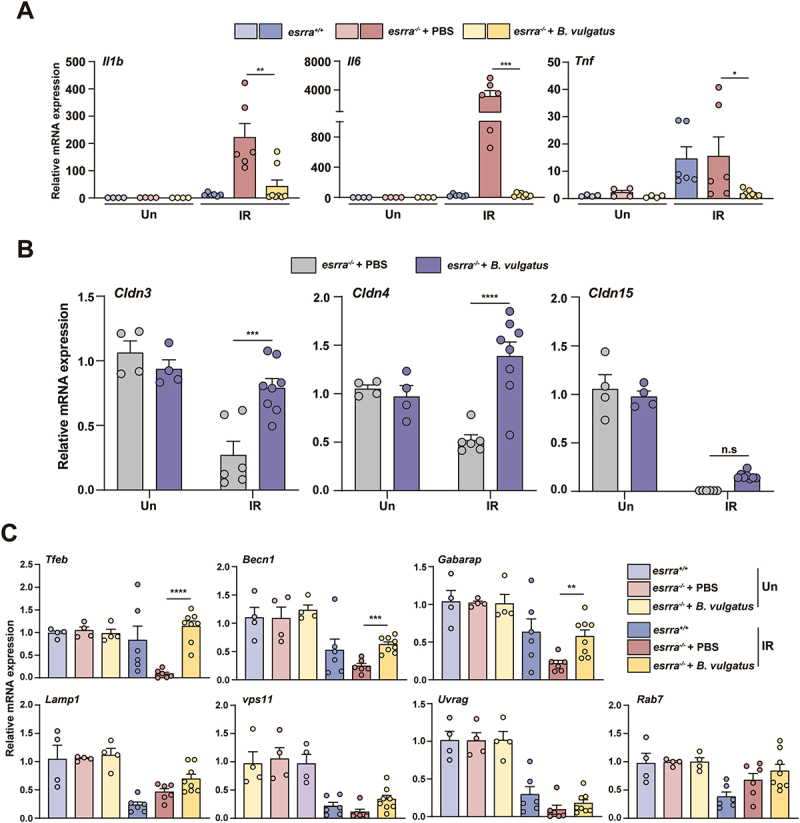
*Bacteroides vulgatus* restored the expression of inflammatory cytokine, tight junction protein, *tfeb* pathway genes in *esrra*^-/-^ mice postirradiation. (A) quantitative PCR analysis of the mRNA levels of proinflammatory cytokines (*Il1β, Il6, and Tnf*) in jejunal tissues from *esrra*^-/-^ mice treated with *B. vulgatus* or PBS for 4 weeks before 17 Gy WAI (*n* > 4 per group). (B) quantitative PCR analysis of tight junction complex protein mRNA levels (*Cldn3, Cldn4, and Cldn15*) in jejunal tissues from *esrra*^-/-^ mice given *B. vulgatus* or PBS for 4 weeks before 17 Gy WAI (*n* > 4 per group). (C) quantitative PCR analysis of the mRNA levels of *Tfeb* and its downstream genes (*Vps11, Uvrag, Becn1, Gabarap, Lamp1, and Rab7*) in jejunal tissues from *esrra*^-/-^ mice treated with *B. vulgatus* or PBS for 4 weeks before 17 Gy WAI (*n* > 4 per group). *p < 0.05, **p < 0.01, ***p < 0.001, ****p < 0.0001, n.s.: not significant. one-way ANOVA (A,B and C). The data are shown as the means ± SEMs from two independent experiments conducted in triplicate. Un, untreated. IR, irradiated.

In addition, an in vivo intestinal permeability assay was performed using fluorescein isothiocyanate (FITC)-dextran on day 5 after WAI. As shown in Figure 4F, serum levels of FITC-dextran were significantly greater in PBS-treated *esrra*^*-/-*^ mice after WAI, indicating decreased intestinal barrier function compared to that in *B. vulgatus*-treated *esrra*^*-/-*^ mice. Similarly, the mRNA levels of proinflammatory cytokines were lower in the small intestines of the *B. vulgatus*-treated group than in those of the PBS-treated group on day 5 of WAI exposure (Figure 5A). Compared with those in the PBS-treated group, the mRNA levels of tight junction complex proteins in the B. vulgatus-treated group were significantly restored (Figure 5B). These results suggest that pretreatment with *B. vulgatus* can significantly reduce radiation-induced GI toxicity by improving survival rates, reducing weight loss and inflammation, and enhancing the integrity of the intestinal barrier.

In previous research, we observed decreased expression of *Tfeb* under stress conditions in *esrra*^*-/-*^ mice.^18^ Therefore, we investigated changes in *Tfeb* expression 5 days after WAI. We found that *Tfeb* mRNA expression was decreased in *esrra*^*-/-*^ mice compared to that in *esrra*^*+/+*^ mice after WAI and was restored when *B. vulgatus* was administered (Figure 5C). Furthermore, we assessed the expression of several *Tfeb* downstream genes, including *Vps11, Uvrag, Becn1, Gabarap, Lamp1, and Rab7*, after WAI. Notably, the expression of *Becn1* and *Gabarap* was restored to levels similar to those of the wild type when *B. vulgatus* was administered (Figure 5C).

### Association of ESRRA expression with increased severity of radiation-induced proctitis: clinical insights from the rectal tissues of patients

**Figure 6.**
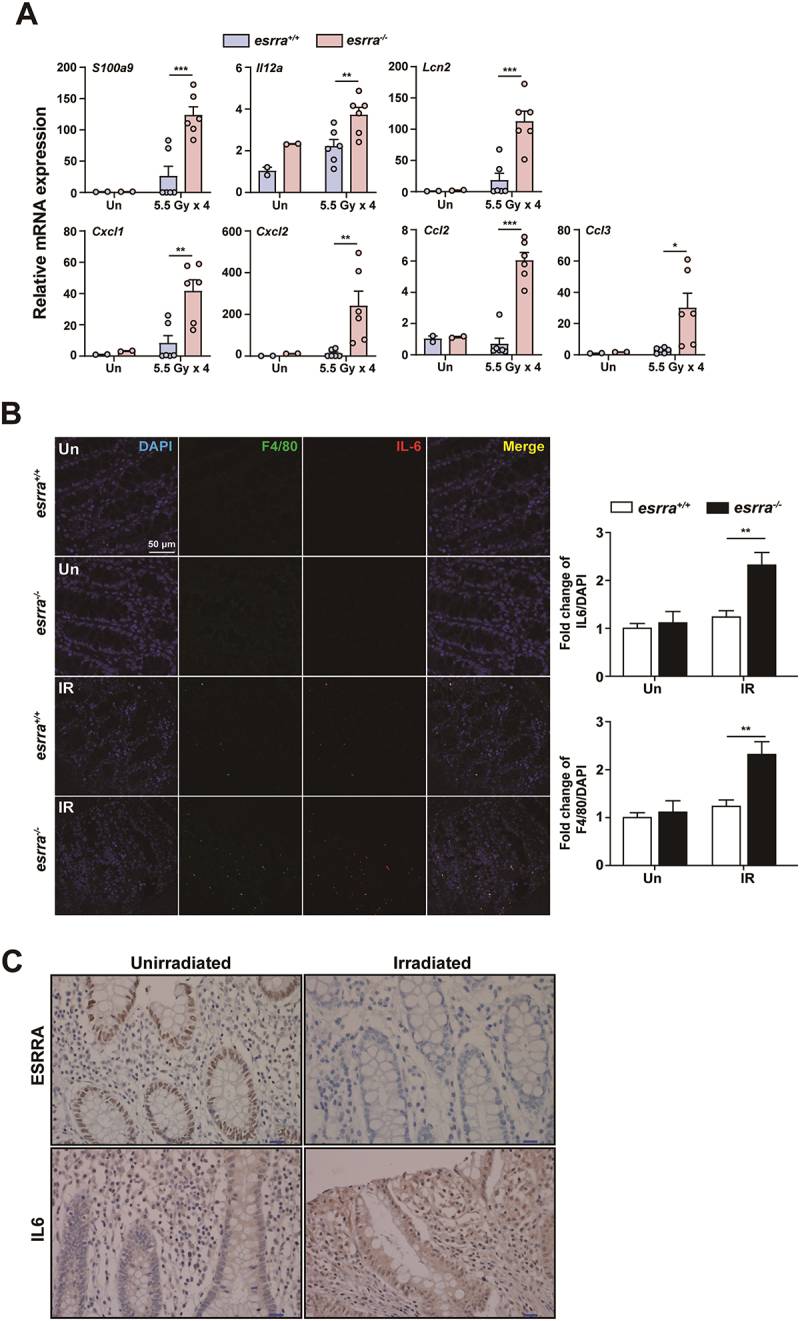
ESRRA expression and inflammation in radiation-induced proctitis in mice and patients. (A) quantitative PCR analysis of inflammatory cytokine and chemokine mRNA levels (*S100a9, Il12a, Lcn2, Cxcl1, Cxcl2, Ccl2, and Ccl3*) in rectal tissues from *esrra*^+/+^ and *esrra*^-/-^ mice following 5.5 Gy, 4 times rectal irradiation (*n* > 2 per group). (B) representative immunofluorescence images and quantitative analysis showing IL-6 (red) and ADGRE1/F4/80 (green) protein levels in rectal tissues from *esrra*^+/+^ and *esrra*^-/-^ mice postirradiation. Scale bar = 50 µm. (C) representative immunohistochemistry images of ESRRA and IL-6 protein levels in nonirradiated and irradiated rectal tissues from patients. Scale bar = 50 µm. *p < 0.05, **p < 0.01, ***p < 0.001. two-way ANOVA (A and B). The data are shown as the means ± SEMs from two independent experiments conducted in triplicate. Un, untreated. IR, irradiated.

To investigate the potential role of ESRRA in alleviating radiation-induced proctitis, we assessed the mRNA expression levels of inflammatory cytokines and chemokines. The mRNA expression levels of inflammatory cytokines and chemokines, including *S100a9, Il12a, Lcn2, Cxcl1, Cxcl2, Ccl2*, and *Ccl3*, were significantly increased in *esrra*^*-/-*^ mice in a rectal proctitis model (Figure 6A). Furthermore, IL-6 expression and macrophage infiltration were increased after rectal irradiation (Figure 6B). Notably, IL-6 and F4/80 protein levels were significantly increased in *esrra*^*-/-*^ mice (Figures 6B and S6A). These data suggest that *esrra* deficiency leads to increased severity of rectal inflammation, including increased IL-6 expression and macrophage infiltration.

Based on previous findings related to the pathogenesis of radiation-induced proctitis, we investigated the expression of ESRRA protein in the rectal tissues of patients to determine its clinical significance. IHC analysis of nonirradiated and irradiated rectal tissue samples revealed that compared with nonirradiated epithelial cells, irradiated epithelial cells exhibited weaker nuclear staining for ESRRA (Figure 6C). The protein expression of IL-6 was also increased in irradiated colonic mucosa, suggesting an inflammatory response associated with radiotherapy (Figure 6C). To assess the potential impact of ESRRA expression, we examined the rectal tissues using IHC in a cohort of 36 irradiated cancer patients, Notably, patients with reduced ESRRA expression experienced a higher incidence of moderate to severe diarrhea during radiotherapy, underscoring ESRRA’s potential role in mitigating radiation-induced gut damage (Table 1). Together, these results suggest that ESRRA may play a protective role in radiation-induced proctitis, potentially serving as a biomarker for predicting radiation induced gastrointestinal toxicity.

**Table 1.** Association between ESRRA protein expression and acute diarrhea in 36 patients with locally advanced rectal cancer treated with preoperative chemoradiotherapy.This table presents the association between ESRRA protein expression and diarrhea in a cohort of 36 patients with locally advanced rectal cancer who underwent preoperative chemoradiotherapy. Patients were classified into low ESRRA expression and high ESRRA expression groups, and radiation induced diarrhea grades (grade 0–1 vs. Grade 2–4) were compared between these two groups. Pearson’s chi-square test was used to evaluate the statistical significance of the association between ESRRA expression and diarrhea grade.

Toxicity and Grade	ESRRA low expression n (%)	ESRRA high expression n (%)	P value*
Diarrhea			0.0113
Grade 2–4	8 (50.0%)	2 (10%)	
Grade 0–1	8 (50.07%)	18 (90%)	*

*Pearson’s chi-square test.

## Discussion

Radiation-induced GI toxicity is a significant obstacle in cancer therapy involving abdominal radiotherapy and in the context of nuclear accidents.^3^ The main symptoms of this condition, i.e., nausea, vomiting, diarrhea, and abdominal pain, degrade quality of life and limit the potential therapeutic dosage of radiation that can be safely administered.^26^ Although high-precision radiation therapies such as IMRT and IGRT deliver a localized distribution of ionizing energy to cure cancers in patients, inevitable radiation exposure to neighboring normal tissue leads to GI toxicity in patients treated with abdominal or pelvic radiation therapy. Furthermore, the current incidence of patients who have long-term radiation-induced GI toxicity exceeds that of patients with IBD, indicating that more studies on radiation biology are needed.^3,27^

Extensive research has been conducted on the function of ESRRA, due to its transcriptional activation of gene sets involved in mitochondrial metabolism and oxidative phosphorylation.^11,28,29^ Additionally, previous studies have shown that ESRRA may be involved in cellular homeostasis which may regulate inflammation, autophagy, and other biological responses, highlighting its potential as a novel therapeutic target in various inflammatory diseases.^17,18,30–32^ Our data reveals ESRRA is crucially involved in mediating the effects of radiation-induced GI inflammation, demonstrating that deficiency of ESRRA may exacerbate intestinal injury, inflammatory cytokine production, and gut microbiota dysbiosis. Conversely, resveratrol, a known ESRRA agonist, demonstrates a potent ability to improve survival rates and attenuate radiation-induced inflammation. Consistent with other studies that emphasize the importance of ESRRA in various stress and damage response contexts, the present study revealed that ESRRA expression is crucial for the intestinal response to radiation stress.^17,33^ Taken together, these results strongly suggest that ESRRA is essential for the innate immune response to a variety of stresses and contributes to maintaining GI integrity following radiation exposure, further highlighting the potential of enhancing this pathway as a strategy for mitigating radiation-induced intestinal injury.

In this study, we used the WAI irradiation mouse model that mimics human disease phenotypes to systematically assess the role of ESRRA in ameliorating radiation damage. In this mouse models, we observed that ESRRA ablation significantly worsened radiation-induced GI toxicity. Following WAI, the expression levels of Il6 and Ccl2 were significantly higher in esrra-/- mice compared to WT mice, indicating enhanced inflammatory responses. However, other cytokines, such as *Il1β, Tnf*, and *Cxcl2*, did not show significant differences, in contrast to our previous findings in DSS-induced colitis. This inconsistency suggests that the differential inflammatory response may arise under different experimental conditions. DSS induces intestinal inflammation through damaging to the epithelial monolayer lining the large intestine allowing the dissemination of bacteria and their products into underlying tissue.^34^ In contrast, radiation-induced toxicity primarily arises from DNA damage, vascular damage and oxidative stress, which results in a more concentrated and tissue-repair-driven inflammatory response.^10^ Further studies are required to elucidate the potential mechanisms in which ESRRA acts to regulate inflammatory responses under various stress conditions.

Our previous research confirmed that ESRRA is involved in regulating DSS-induced colitis and infections through autophagy activation and TFEB nuclear translocation.^18,33^ Recent study demonstrated a reciprocal regulatory relationship between TFEB and ESRRA, where TFEB directly binds to the ESRRA promoter to enhance its transcription, while ESRRA promotes TFEB expression by regulating upstream signaling pathways involved in autophagy and lysosomal biogenesis.^35^ This bidirectional regulation forms a feedback loop that coordinates stress responses, highlighting the functional interplay between these two genes in maintaining cellular homeostasis.^35^ Consistent with this, we observed significant downregulation of TFEB and downstream gene expressions in ESRRA-null intestinal epithelial cells, which led to impaired autophagy and disrupted autophagy flux and defective cellular clearance.^18^ In agreement with our previous study, we showed that WAI further reduced the *Tfeb* expression in *esrra*^*-/-*^ mice. However, administration of *B. vulgatus* restored *Tfeb* expression in *esrra*^*-/-*^ mice to levels comparable to *esrra*^*+/+*^ mice. This restoration was accompanied by the upregulation of key *Tfeb*-regulated autophagy genes, including *Becn1* and *Gabarap*, which are critical for autophagosome formation and elongation. These results imply that ESRRA-mediated regulation of *Tfeb* is involved in DSS-induced colitis and radiation-induced intestinal injury, further supporting its role in maintaining intestinal homeostasis in response to diverse stress state.

Numerous studies have shown that diet, nutrients, pharmacologic factors, and other stimuli play dominant roles in modulating the composition of the gut microbiome.^36^ In addition, there is growing interest in the impact of host factors on shaping the gut microbiota^37^ The gut microbiota of *esrra*^*-/-*^ mice is significantly more diverse than that of *esrra*^*+/+*^ mice, and ESRRA ameliorates DSS-induced colitis by remodeling the gut.^18^ Notably, a series of studies of twins reported that host factors such as specific genes, innate immune sensors, antimicrobial peptides, the mucus barrier, and secretory IgAs (sIgA) directly affect the gut microbiota. Key pathway molecules in innate immune sensors include Toll-like receptors (TLRs) and NOD-like receptors. TLR2 impacts *Helicobacter* abundance, while TLR4 influences Fusobacteria, Proteobacteria, and Bacteroidetes populations.^38–41^ Antimicrobial peptides such as defensin alpha 5 (DEFA5) and resistin-like molecule β (RELMβ) affect microbial composition by targeting bacterial structures.^42,43^ Secretory Immunoglobulin A binds bacteria, enhancing beneficial microbial colonization while limiting pathogens.^44^ Claudin-3, a tight junction protein, plays a critical role in maintaining normal gut microbiota and inflammatory responses in IBD patients.^45^ Although the precise mechanisms by which *B. vulgatus* reverses gut pathologies in *esrra*^*-/-*^ mice are currently unknown, our data strongly suggest that this species can be useful for potential therapeutics against radiation-induced colitis.

Gut dysbiosis is a critical factor in the pathogenesis of radiation-induced intestinal injury.^46^ A decrease in the abundance of Firmicutes and Bacteroidetes after radiotherapy suggests that repopulation of these microorganisms may play protective roles in reconstituting the structure of the gut microbiota, thereby alleviating radiation-induced intestinal injury.^47,48^ The probiotic *Bacteroides fragilis* promotes epithelial cell proliferation in the small intestine, stem cell regeneration, goblet cell secretion, and tight junction repair through the STAT3 signaling pathway in mouse models of radiation-induced intestinal injury.^49^ Our study focused on elucidating the interactions among ESRRA, the gut microbiota, and radiation-induced GI toxicity. We found that WAI induced significant alterations in the gut microbiota of both *esrra*^*+/+*^ and *esrra*^*-/-*^ mice; however, these changes were more pronounced in *esrra*^*-/-*^ mice, which exhibited greater susceptibility to radiation-induced colitis than that *esrra*^*+/+*^ mice. Given that *B. vulgatus* is abundant in the intestines of *esrra*^*+/+*^ mice, we treated *esrra*^*-/-*^ mice with *B. vulgatus* and found that the intestinal pathologies were reversed.^18^ Interestingly, *esrra*^*-/-*^ mice administered *B. vulgatus* exhibited a greater reduction in *Tnf* expression than *esrra*^*+/+*^ mice, suggesting additional mechanisms beyond ESRRA. The protective role of *B. vulgatus* in intestinal inflammation has been reported in earlier studies, which have identified its role in immune balance through the modulation of gut microbiota composition, short-chain fatty acid (SCFA) production, and inflammasome activity.^50,51^ Furthermore, *Tfeb* and its downstream autophagy-related genes (*Becn1 and Gabarap*), which exhibited lower expression in *esrra*^-/-^ mice post-WAI, were recovered after *B. vulgatus* administration suggesting that its protective effects were mediated by microbiota modulation. These findings indicate that the loss of ESRRA expression markedly increases susceptibility to radiation induced GI toxicity by both reshaping the microbiota and directly modulating innate immune responses.

To strengthen our findings, we conducted rectal proctitis mouse model experiment, which is similar with the irradiation field applied in rectal cancer patients. In this experiment setting, *esrra*^-/-^ mice exhibited an exacerbated inflammatory response, which resembled the that of the WAI model. Consistently, IHC analysis of rectal tissues from irradiated patients revealed reduced ESRRA nuclear staining and increased IL-6 levels, further supporting for an inverse relationship between ESRRA expression and radiation-induced inflammation. Clinical data further support our hypothesis regarding the importance of ESRRA in mitigating radiation-induced gastrointestinal toxicity. Taken together, both mouse and human data revealed the translational potential of ESRRA as a biomarker and therapeutic target for radiation-induced gut injury.

In summary, our study demonstrates that ESRRA plays a crucial role in protecting against radiation-induced gastrointestinal toxicity via regulation inflammatory responses, maintenance intestinal homeostasis, and modulation gut microbiota composition. Notably, we identified *B. vulgatus* was critical in restoring *Tfeb* expression, enhancing autophagy-related pathways, and alleviating radiation-induced intestinal injury. These findings suggest that gut enrichment of *B. vulgatus* could offer a new option to combat the radiation induced gastrointestinal complications. Furthermore, human tissue and clinical data revealed that reduced ESRRA expression was associated with increased IL-6 levels and a higher incidence of moderate to severe radiation-induced diarrhea. Given the strong link between ESRRA and radiation-induced GI toxicity, our data suggests that enhancing its expression may be an effective approach to diminish pathological inflammatory responses, thereby limiting radiation-induced intestinal toxicity. Future studies are needed to develop pharmacological agents to upregulate ESRRA and to design patient-centric microbiome studies that could improve therapeutic protocols for managing radiation-induced side effects.

## Materials and methods

### Ethics approval and consent to participate

Mice were treated in accordance with the guidelines of the Institutional Animal Care and Use Committee, Chungnam National University School of Medicine, Daejeon, Korea (2019012A-CNU-197).Nonirradiated and irradiated rectal tissues for this study were collected from the institutional review board (IRB) of Chungnam National University Hospital (IRB no. 2020–03–105) and analyzed following the study protocol. Informed consent was exempted from the IRB due to the retrospective design. All patients had provided prior general consent at the time of biopsy or surgery, allowing their specimens to be used for future research purposes. As this study was retrospective in nature and used anonymized data and archived specimens, the IRB approved the study with a waiver of specific informed consent.

The human studies used formalin-fixed, paraffin-embedded (FFPE) rectal tissue samples obtained from 36 patients with locally advanced rectal cancer who underwent preoperative chemoradiotherapy. In addition to tissue samples, anonymized clinical data were collected, including patient sex, ESRRA expression status, and the severity of radiation-induced diarrhea (graded using CTCAE v5.0). These data were used to evaluate the clinical correlation between ESRRA expression and gastrointestinal toxicity.

### Animals

Eight- to 12-week-old *esrra*^*+/+*^ and *esrra*^*-/-*^ mice, matched for sex, were used in this study. All mice were housed under specific pathogen-free conditions. Experiments were conducted using littermates from heterozygous mice separated by sex and genotype (single cage).

For treatment of *Bacteroides vulgatus*, the type strain of *B. vulgatus* (JCM 5826) was cultured under anaerobic conditions using Gifu Anaerobic Medium (GAM) broth or agar supplemented with 1.5% Bacto agar (214010; BD Difco) at 37°C. The bacteria were harvested during the logarithmic growth phase and resuspended in PBS to a concentration of 10^8^ CFU/ml for gavage. Briefly, all mice were gavaged twice a week and allowed to feed freely with common food and water under the same environmental conditions. Control mice received 200 μL of PBS by oral gavage twice a week for 4 weeks. The irradiation experiment was performed 1 week after *B. vulgatus* or treatment with PBS.

### Irradiation

External beam radiotherapy was delivered at Chungnam National University Hospital in the Radiation Oncology Department using an Elekta Synergy Platform linear accelerator (Elekta, Crawley, UK). The dose rate was 3 Gy/min. Male mice were exposed to a single dose of 17 or 17.5 Gy. IR was delivered using lead shielding so that the whole abdomen was irradiated, and the other parts of the mouse were shielded.

The radiation-induced proctitis model was adapted from a previous study and involved the use of a high-dose-rate brachytherapy system with an Ir-192 source.^52^ Two mice at a time were anesthetized and placed with lead shielding between them to reduce scatter radiation, and a cylindrical polystyrene applicator was inserted into the rectum up to 2 cm. The radiation dose was focused on a cylinder-shaped target 3 mm from the applicator’s surface and calculated using Varian BrachyVision software. A 24-hour interval was maintained between treatment fractions to allow for the repair of sublethal damage. The control mice received a sham treatment involving the same procedure without actual radiation exposure.

### Histology

The small and large intestines were dissected, flushed with phosphate-buffered saline (PBS), and linearized longitudinally. The tissue was Swiss-rolled, and fixed in 10% neutral buffered formalin, dehydrated in ethanol, and then embedded in paraffin. Embedded tissues were sectioned at 6 mm and stained with hematoxylin and eosin (H&E) under a light microscope.

### Immunohistochemistry

For immunostaining, we utilized a 3,3′-diaminobenzidine peroxidase substrate kit (DAKO Cytomation, Inc., Carpinteria, CA, USA). Briefly, sections from paraffin blocks with a thickness of 3 µm were deparaffinized in xylene and rehydrated in graded alcohol. After antigen retrieval by heating in a pressure cooker in 10 mM sodium citrate buffer (pH 6.0) for 4 min, endogenous peroxidase blocking (0.03% H_2_O_2_) was performed for 10 min at room temperature. The tissue sections were incubated at room temperature for 30 min with a monoclonal rabbit antibody directed against ESRRA (1:800, Cell Signaling Technology [CST], 13826S), a monoclonal rabbit antibody directed against IL-6 (1:400, Santa Cruz Biotechnology [SCBT], sc -57315), and a monoclonal mouse CD68 (clone PG-M1, Thermo Fisher Scientific). After washing, the sections were treated with 100 µl of secondary antibody [EnVision + Single Reagents horseradish peroxidase (HRP); cat. no. K4003; Dako; Agilent Technologies, Inc.] for an additional 20 min at room temperature and developed for 15 min using 3,3’-diaminobenzidine (DAB) as the substrate chromogen. Slides were counterstained with Meyer’s hematoxylin for 30 sec and mounted. All slides were thoroughly rinsed several times with TBS-T between each step. The results were examined separately by 2 pathologists who were blinded to the patients’ clinicopathological details.

### Immunofluorescence

Immunofluorescence staining was performed on 3-µm thin sections of mouse small intestine and rectal tissues. The tissue sections were incubated at room temperature for 30 min with a mouse monoclonal antibody against IL-6 (1:50; Santa Cruz Biotechnology [SCBT], sc -57315) and a rat monoclonal antibody against ADGRE1/F4/80 (1:50; SCBT, sc -52664). Then, the sections were treated with 100 µl of the following secondary antibodies: Alexa Fluor 488-conjugated goat anti-rat IgG (H + L) (1:100; A-11006) and Alexa Fluor 594-conjugated goat anti-rat IgG (H + L) (1:100; A-11005). The nuclei were stained using 4′,6-diamidino-2-phenylindole (DAPI, Sigma‒Aldrich, D9542) for 1 min. A confocal laser-scanning microscope (Zeiss, Germany) was used to capture immunofluorescence images.

### RNA extraction and quantitative real-time PCR (qPCR) analysis

Total RNA from murine jejunal and rectal tissues was isolated using TRIzol reagent (Invitrogen 15596026), and cDNA was synthesized using Superscript II Reverse Transcriptase (Elpisbio, EBT-1515C). qPCRs were carried out using QuantiNova SYBR Green PCR Kits (Qiagen 208056) in the Real-time PCR Cycler Rotor-Gene Q 2plex system (Qiagen 9001620). The amplification process involved 40 cycles, each consisting of a denaturation step at 95°C for 10 seconds and an annealing step at 60°C for 30 seconds. To analyze the qPCR data, we utilized the 2ΔΔCt method for relative quantification, employing mouse β-actin as an internal control gene. The results are presented as relative fold changes. The specific primer sequences utilized are listed in Table 1.

### Western blot

Jejunal tissues were homogenized in radioimmunoprecipitation assay (RIPA) buffer composed of 50 mM Tris-HCl (pH 7.5), 2 mM EDTA, 150 mM NaCl, 0.1% sodium dodecyl sulfate (SDS; Sigma‒Aldrich, R0278), 1% sodium deoxycholate [Life Technologies, 89904], and 1% Triton X-100 (Sigma, T8787). RIPA buffer supplemented with protease inhibitor cocktail (Roche, 11836153001) and phosphatase inhibitor cocktail (Sigma‒Aldrich, P5726) was used. Protein extracts were boiled in 1× SDS sample buffer and processed for immunoblotting analysis. These extracts were then separated by SDS‒polyacrylamide gel electrophoresis and subsequently transferred to polyvinylidene difluoride membranes (Millipore).

The membranes were blocked in 5% nonfat milk in PBST (3.2 mM Na2HPO4, 0.5 mM KH2PO4, 1.3 mM KCl, 135 mM NaCl, 0.05% Tween 20 [Sigma‒Aldrich, P1379], pH 7.4) for 1 h and incubated with the following primary antibodies: anti-ESRRA (13826S), anti-phospho-MAPK/ERK (9101 s), anti-MAPK/ERK (9102s) and anti-PTGS2/COX-2 (12282S) from CST; and anti-ACTB/β-actin (sc -47778) and anti-IL-6 (sc -57315) from SCBT. Following incubation with the appropriate secondary antibodies, immunoreactive protein bands were visualized using an enhanced chemiluminescence (ECL) reagent (Millipore, WBKLS0500) in a UVitec Alliance mini-chemiluminescence device (UVitec, UK).

### Measurement of intestinal permeability

Mice were irradiated with a single dose of 17 Gy to the whole abdomen. Five days later, all the mice were starved overnight the day before sacrifice. Mice were fed FITC-dextran (Sigma‒Aldrich 46944) dissolved in PBS (40 mg/100 g body weight) via oral gavage 1 h before sacrifice. Whole blood was collected by cardiac puncture under full anesthesia (Avertin, 200 mg/kg, i.p.), immediately before euthanasia via CO₂ inhalation. This procedure was performed during terminal sample harvesting and followed humane endpoints as defined in the approved protocol. Sera were isolated from a yellow blood tube and centrifuged at 2500 rpm for 7 min. Diluted FITC-dextran was used for a standard curve. The harvested supernatant was diluted with the appropriate factor and measured by a fluorometer at an excitation wavelength of 485 nm and emission wavelength of 528 nm.

### Fecal DNA extraction and 16S rRNA gene sequencing

Fecal samples were collected from the mice, and DNA for metagenomic analysis was extracted using a QIAamp DNA Stool Mini Kit (51504; Qiagen) according to the manufacturer’s instructions. The hypervariable regions of the 16S rRNA gene (V3–V4) were amplified by PCR using bacterial universal primers: 341F (5′-TCGTCGGCAGCGTCAGATGTGTATAAGAGACAGCCTACGGGNGGCWGCAG-3′) and 805 R (5′-GTCTCGTGGGCTCGGAGATGTGTATAAGAGACAGGACTACHVGGGTATCTAATCC-3′). PCR was performed using C1000 thermal cyclers (Bio-Rad) according to the Illumina 16S Metagenomic Sequencing Library Preparation manual (Part # 15044223 Rev. B) using the following conditions: an initial denaturation step at 95°C for 3 min followed by 24 cycles of 95°C for 30 s, 55°C for 30 s, and 72°C for 30 s, with a final step at 72°C for 5 min. Triplicate PCR reactions with the same DNA template were pooled and purified using a QIAquick PCR Purification kit (28106; Qiagen). Purified amplicons were sequenced on an Illumina MiSeq platform using a 2 × 300 bp reagent kit for paired-end sequencing by Macrogen Inc. (Seoul, South Korea).

The amplicon sequencing data were imported into and analyzed on the QIIME2 (version 2023.07),^53^ and imported paired reads were trimmed using cutadapt.^54^ After trimming, sequences were denoised using the DADA2 algorithm^55^ in the QIIME2 package and trimmed based on the sequencing quality plot (median quality score > 30) using the parameters (–p-trim-left-f [0] –p-trim-left-r [0] –p-trunc-len-f [267] –p-trunc-len-r [200]). Low frequency and low prevalent amplicon sequence variants (ASVs) were removed (–p-min-frequency [20] –p-min-samples [2]), and data were rarefied to the lowest read count using the parameter (–p-sampling-depth [12000]). ASVs were taxonomically classified by classify-sklearn plugin with a pre-trained Naïve Bayes classifier against the SILVA database v138.1^56^ (99% similarity clustered V3-V4 region 16S rRNA reference sequence). The sequences of the ASVs belonging to the species *B. vulgatus* were identified through EZbiocloud database.^23^ Alpha diversity (within-sample) was estimated using number of observed ASVs, Shannon diversity index, Pielou’s evenness index, and Faith’s phylogenetic diversity whole tree. Beta diversity was calculated based on unweighted and weighted UniFrac distance by principal coordinates analysis (PCoA). PCoA results and relative abundance table were imported to R (version 4.2.3) using qiime2R package (version 0.99.6; https://github.com/jbisanz/qiime2R) and read.table function, respectively. PCoA plots and relative abundance bar plots were generated using ggplot2 package (version 3.4.2).^57^

### Quantification of *B*. *vulgatus* abundance using qPCR

Extracted fecal DNA was used for *B*. *vulgatus* quantification analysis. qPCR was performed using Dyne qPCR 2X PreMIX (DYRT1202; DYNE BIO) following the manufacturer’s instruction. Specific primers for *B*. *vulgatus* and total bacteria were used as previously reported.^25^ The specific primer sequences are listed in Table 2.

**Table 2.** Primers used in this study.

Genes	Primer	Sequences
*Actin*	Forward Reverse	5′- TGG CAA AGT GGA GAT TGT TGC C −3′ 5′- AAG ATG GTG ATG GGC TTC CCG −3′
*Tnf*	Forward Reverse	5′- AGC CGA TGG GTT GTA CCT TG −3′ 5′- ATA GCA AAT CGG CTG ACG GT −3′
*Il6*	Forward Reverse	5′- ACA AAG CCA GAG TCC TTC AGA −3′ 5′- TGG TCC TTA GCC ACT CCT TC −3′
*Il1b*	Forward Reverse	5′- TGG ACC TTC CAG GAT GAG GAC A −3′ 5′- GTT CAT CTC GGA GCC TGT AGT G −3′
*S100a9*	Forward Reverse	5′- ACC ACC ATC ATC GAC ACC TTC −3′ 5′- AAA GGT TGC CAA CTG TGC TTC −3′
*Lcn2*	Forward Reverse	5′- TGA GTG TCA TGT GTC TGG GC −3′ 5′- AAC TGA TCG CTC CGG AAG TC −3′
*Ccl2*	Forward Reverse	5′- ACT CAA GCC AGC TCT CTC TT −3′ 5′- TTC CTT CTT GGG GTC AGC AC −3′
*Ccl3*	Forward Reverse	5′- CAT ATG GAG CTG ACA CCC CG −3′ 5′- GAG CAA AGG CTG CTG GTT TC −3′
*Cxcl1*	Forward Reverse	5′- GAC CAT GGC TGG GAT TCA CC −3′ 5′- CGC GAC CAT TCT TGA GTG TG −3′
*Cxcl2*	Forward Reverse	5′- CCC TGC CAA GGG TTG ACT TC −3′ 5′- GCA AAC TTT TTG ACC GCC CT −3′
*Il12a*	Forward Reverse	5′- CAC AAG AAC GAG AGT TGC CTG GCT ACT A −3′ 5′- TAA GGG TCT GCT TCT CCC ACA GGA GGT T −3′
*Tfeb*	Forward Reverse	5′- ACA TAT CAG CTC CAA CCC CG −3′ 5′- CGT TCA GGT GGC TGC TAG AC −3′
*Vps11*	Forward Reverse	5′- ATC GGC AGT CTC TGG CTA ATG C −3′ 5′- GGA CCT TGA TGG CTG TCT CTA C −3′
*Uvrag*	Forward Reverse	5′- GAC TTT GGA ATA ATG CCG GAT CG −3′ 5′- CAG CCC ATC CAG GTA GAC TTT-3′
*Becn1*	Forward Reverse	5′- CAG CCT CTG AAA CTG GAC ACG A −3′ 5′- CTC TCC TGA GTT AGC CTC TTC C −3′
*Gabarap*	Forward Reverse	5′- GGT CCC GGT GAT AGT GGA AAA A −3′ 5′- AAC AAG GCA TCT TCA GCA CG −3′
*Rab7*	Forward Reverse	5′- GAG CGG ACT TTC TGA CCA AGG A −3′ 5′- CAA TCT GCA CCT CTG TAG AAG GC −3′
*Lamp1*	Forward Reverse	5′- CAG CAC TCT TTG AGG TGA AAA AC −3′ 5′- CCA TTC GCA GTC TCG TAG GTG −3′
*Cldn3*	Forward Reverse	5′- GTA CAA GAC GAG ACG GCC AA −3′ 5′- CGT ACA ACC CAG CTC CCA TC −3′
*Cldn4*	Forward Reverse	5′- ACA ACC CTA TGG TGG CTT CC −3′ 5′- TAC ACA TAG TTG CTG GCG GG −3′
*Cldn15*	Forward Reverse	5′- CGT GGG CAA CAT GGA TCT CT −3′ 5′- CCA CGA GAT AGC CAC CAT CC −3′
*Chchd10*	Forward Reverse	5′- CAC TCA GAG CGA CCT AAC CC −3′ 5′- GGA GCT CAG ACC GTG ATT GT −3′
*B. vulgatus*	Forward Reverse	5’ – CGA TTG GTC TGG CAC GTA TG − 3’ 5’ – ACT TCA TTG TCA CGC ACA TTC AT − 3’
All bacteria	Forward Reverse	5’ – TGS TGC AYG GYT GTC GTC A − 3’ 5’ – ACG TCR TCC MCA CCT TCC TC − 3’

## Statistical analysis

GraphPad Prism, v8.0 or v10.2.3 (GraphPad Software, Inc.) were used to analyze the data. The data are presented as the mean ± standard deviation. For all the statistical tests, a probability value (*p* value) of 0.05 or less was indicated with *p < 0.05, **p < 0.01, ***p < 0.001, and ****p < 0.0001. Data with a *p* value less than 0.05 were considered significant. Normality tests (D’Agostino and Pearson omnibus normality test) were carried out, and the results were found to conform to normality. The data were analyzed using two-tailed Student’s t tests or nonparametric tests, as appropriate. For nonparametric tests, the Mann‒Whitney U test was used for comparing two conditions, and one-way ANOVA with Dunn’s multiple comparison test was applied for three or more conditions. The results of alpha diversity were compared using multiple Mann-Whitney U test with multiple comparison tests using Holm-Šídák method. The statistical significance of PCoA was calculated by pairwise PERMANOVA test with 10,000 permutations and pairwise results were shown as *p* value in the plot. For comparisons across multiple groups under different conditions, two-way ANOVA with Bonferroni correction was used to compare the data from each *esrra*^*-/-*^ condition with the corresponding e*srra*^*+/+*^. For patient samples, the relationship between ESRRA protein expression and diarrhea grade (Grade 0–1 vs. Grade 2–4) was evaluated using Pearson’s chi-square test.

## Availability of data and materials

The 16S rRNA gene amplicon sequencing data have been deposited in the NCBI SRA database under accession number PRJNA1139376.

## Supplementary Material

Supplemental Material

## Abbreviations

GI: Gastrointestinal
IMRT: Intensity-modulated radiotherapy
IGRT: Image-guided radiotherapy
ESRRA: Estrogen-related receptor alpha
IBD: Inflammatory bowel disease
DSS: Dextran sulfate sodium
IHC: Immunohistochemistry
WAI: Whole-abdominal irradiation
RSV: Resveratrol
I.P: Intraperitoneal injection
qPCR: Quantitative polymerase chain reaction
PBS: Phosphate-buffered saline
OTU: Operational taxonomic unit
ASV: Amplicon sequence variant
PCoA: principal coordinates analysis
FITC: Fluorescein isothiocyanate
HRP: Horseradish peroxidase
DAB: 3,3′-diaminobenzidine
DAPI: 4′,6-diamidino-2-phenylindole
ECL: Enhanced chemiluminescence
TLR: Toll-like receptor
NOD: Nucleotide-binding oligomerization domain
DEFA5: Defensin alpha 5
RELMβ: Resistin-like molecule β
IRB: Institutional review board
H&E: Hematoxylin and eosin
