# Supplementary material for: Estrogen-related receptor alpha mitigates radiation-induced bowel injury through gut enrichment of Bacteroides vulgatus
Source: Gut Microbes. 2025 Aug 19;17(1):2541020. doi: 10.1080/19490976.2025.2541020 (PMC12366819; doi:10.1080/19490976.2025.2541020)
Supplement: Supplemental Material [file KGMI_A_2541020_SM2493.zip › 2541020/Suppl_clean_2_revised__1_.docx]

**Supplemental file for:**

**Estrogen-related receptor alpha mitigates radiation-induced bowel injury through gut enrichment of *Bacteroides vulgatus***

**Running Title:** Estrogen-related receptor alpha protects against radiation bowel damage by enhancing gut *Bacteroides vulgatus*

**Supplementary Figures and Figure Legends**

**
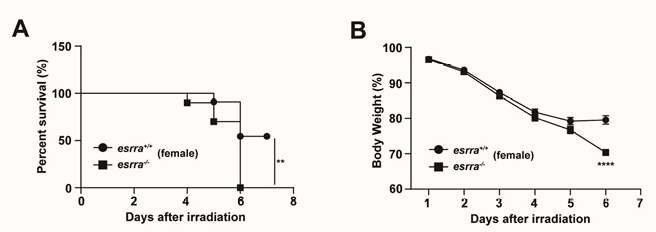
**

**Figure S1.** ESRRA expression is critical for protecting against radiation-induced gastrointestinal toxicity. (A) Survival rates of *esrra*^+/+^ and *esrra*^-/-^ female mice postirradiation with 17 Gy WAI. The data are presented as Kaplan‒Meier survival curves (n > 5 per group). (B) Body weight changes in *esrra*^+/+^ and *esrra*^-/-^ female mice following 17 Gy WAI (n > 5 per group). ***P < 0.01, ****P < 0.0001.* log-rank (mantel-Cox) test (A) and two-way ANOVA (B). Data shown as means ± SEM from two independent experiments conducted in triplicate.


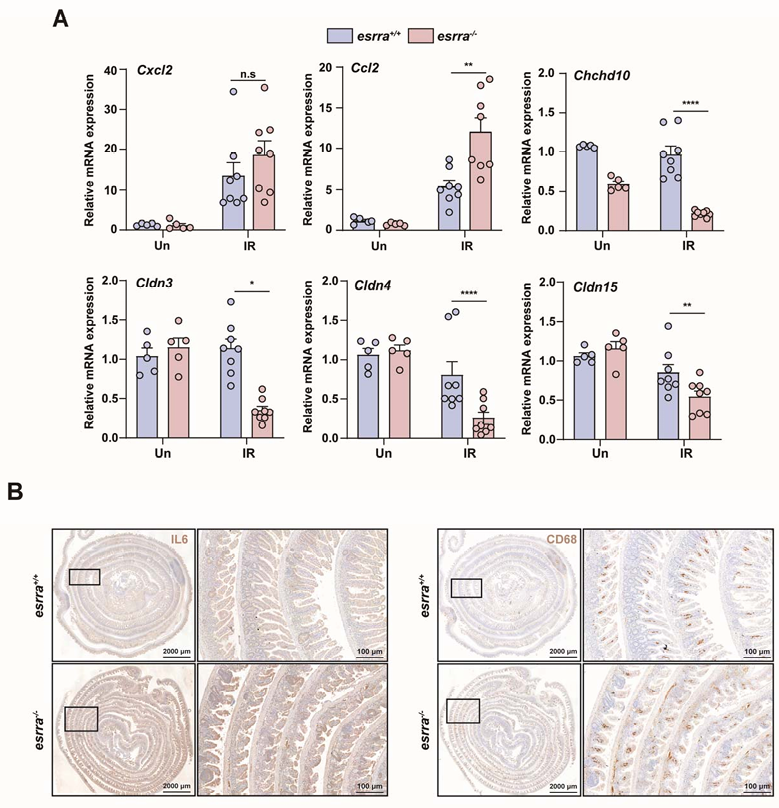


**Figure S2.** ESRRA deficiency increases inflammation and decreases tight junctions in irradiated small intestine tissues. (A) Quantitative PCR analysis of mRNA levels of chemokine (*Cxcl2, and Ccl2*), mitochondrial function (*Chchd10*), and tight junction (*Cldn3, Cldn4, and Cldn15*) genes in jejunum tissues from *esrra*^+/+^ and *esrra*^-/-^ mice following 17 Gy WAI (0 and 5 d of WAI; n > 5 per group). (B) Representative IHC images of the staining showing IL-6 (Left) and CD68 (Right) protein levels in small intestine tissues following 17 Gy WAI. Tissue samples for immunohistochemistry were prepared using the Swiss roll method. **P < 0.05, **P < 0.01, ****P < 0.0001,* ns: not significant. Two-way ANOVA (A). Data shown as means ± SEM from two independent experiments conducted in triplicate. Un, untreated. IR, irradiated.


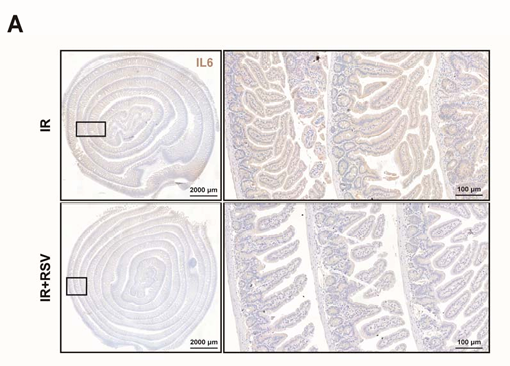


**Figure S3.** The ESRRA agonist resveratrol mitigated radiation-induced inflammation. (A) Representative IHC images of the staining showing IL-6 protein level in small intestine tissues from resveratrol- or PBS-treated *esrra*^+/+^ mice following 17 Gy WAI. Tissue samples for immunohistochemistry were prepared using the Swiss roll method.


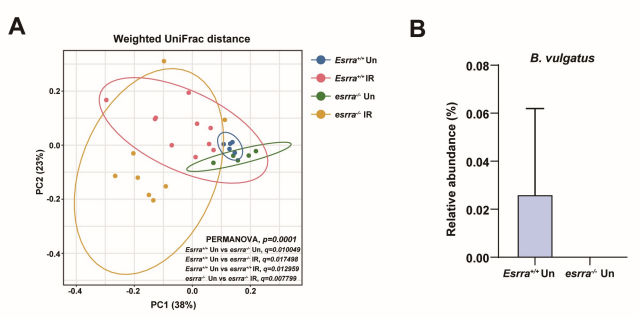


**Figure S4.** Gut microbiota differences between *esrra^+/+^* and *esrra^-/-^* mice before and after WAI. (A) PCoA results based on weighted UniFrac distances of 16S rRNA amplicon sequencing data from *esrra^+/+^* and *esrra^-/-^* mice before and after WAI, respectively. (B) The relative abundance of *Bacteroides* *vulgatus* between *esrra^+/+^* and *esrra^-/-^* mice before WAI (n > 5 per group). Statistical significance was determined by the PERMANOVA with 1000 permutations with pairwise comparison (A) and Mann-Whitney U test (B). The error bars indicate the means ± SEM. Un, untreated. IR, irradiated.


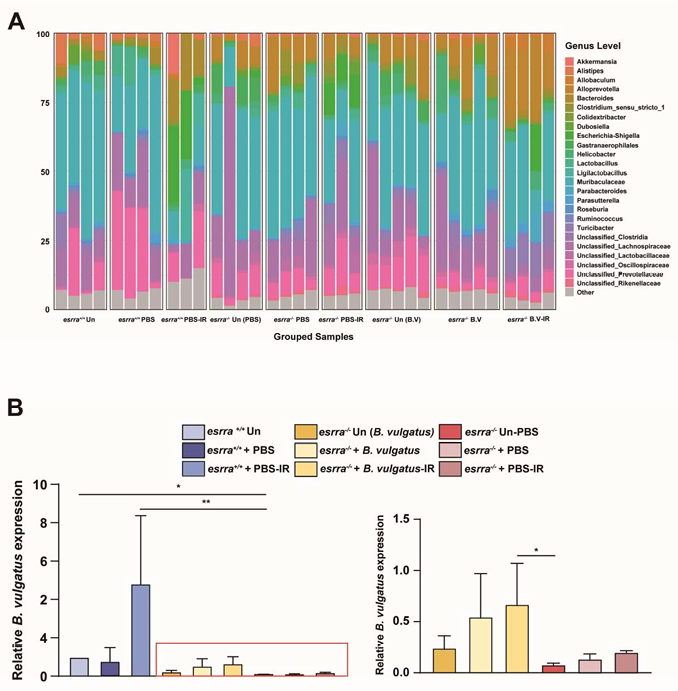


**Figure S5**. Gut microbial composition between *esrra^+/+^* and *esrra^-/-^* mice following *B*. *vulgatus* treatment, before and after WAI. (A) Bar chart showing the relative abundance of major bacterial genera (>1%) (n > 3 per group) using 16S rRNA amplicon sequencing. Taxa with a mean relative abundance of less than 1% across all samples are grouped as Others. (B) Real-time qPCR analysis quantifying the relative abundance of *B. vulgatus* in all experimental groups (n > 3 per group). Statistical significance was determined using the Kruskal-Wallis test with Dunn’s multiple comparisons test (**P* < 0.05, ***P* < 0.01). Error bars indicate the means ± SEM. Un, untreated; BV, *B. vulgatus* treated; IR, irradiated.


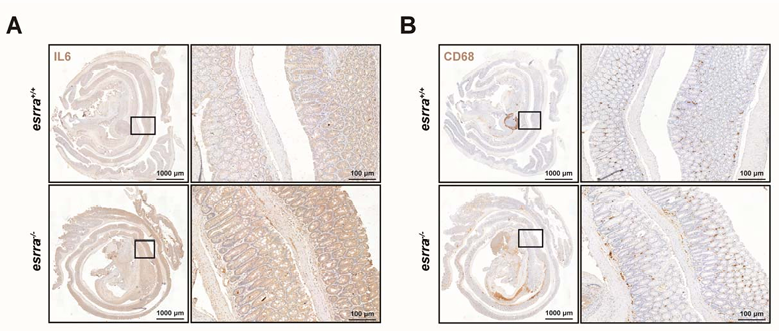


**Figure S6**. ESRRA deficiency increases inflammation in radiation-induced proctitis. Representative IHC images showing IL-6 (Left) and CD68 (Right) protein levels in rectal tissues from *esrra*^+/+^ and *esrra*^-/-^ mice postirradiation. Tissue samples for immunohistochemistry were prepared using the Swiss roll method.
